# Supplementary material for: Enteric Chromosomal Islands: DNA Packaging Specificity and Role of λ-like Helper Phage Terminase
Source: Viruses. 2022 Apr 15;14(4):818. doi: 10.3390/v14040818 (PMC9026076; doi:10.3390/v14040818)
Supplement: Supplementary file 1 [file viruses-14-00818-s001.zip › viruses-1622984-supplementary.pdf]

Table S1. PICI and Phage DNA Recognition Sites and Proteins.

Bacterium<br>[Accession Number]<br>Island name	Rpp/TerS-λ {number or amino acids} [Accession #] (Range)	*cosQ* to bp300. *cosQ* and *cosN* are highlighted in yellow, the Rpp start codon in green, and the Rpp coding sequence continued in grey. For *cos*λ, R3, R2 and R1 are highlighted.
E. Coli CFT073<br>[AAN79974.1]<br>PICI-A	RppA {144} [AE014075]<br>(*rpp* Range:1388373-1388654)<br><br>MIDKACFVSQQEIAEHFKVNRTTIRAWTKQGMPYLNADR<br>GKSGGYHIGHTLLWSSGKSRLEAIRYHVETSALEKIMFA<br>RLLSSERDEYSSEETEHRFDEGLQIYGYSPEDVSKARNK<br>MAGFLAGWRHAVSVRRASMEQSADTEQ	GGGTCCTTTCCGGCATATGGACCCGTTACGGGGCGGCGACCTCGCGGG<br>TTTTCGCTATTTATGACGTTTTTCCGTGAAGGTGACACCACCACCACT<br>TGATTAATATTTAACCATGCAGTTAAGGTAACATTATGATTGATAAAG<br>CTTGTTTTGTAAGTCAGCAGGAAATAGCTGAACATTTCAAGGTTAACA<br>GAACCACTATTCGCGCATGGACCAAACAGGGGATGCCGTATCTTAATG<br>CGGATCGCGGAAAGTCTGGCGGTTATCACATCGGGCATACATTGCTTT<br>GGTCTTCAGGTAAAAGCCGTCTTGAGGCCATCAGATATCACG
E. coli upec-187<br>[NZ_JSLW01000069.1]<br>PICI-B	RppB {146} [WP_033562019.1]<br>(*rpp* Range: = 207846-211846)<br><br>MNNKDCFVSQQEIAEHFKVNRTTIRAWTKQGMPYLDADR<br>GKSGGYHIGHTLFWCMGKSHLDAIEYHGETSALEKIMVA<br>RLISLERDEYFSEETEQRFDNGLQIYGYSPEDVSKARNK<br>MAGFLAGWRHAVAIRREHLQQSVVTEQEN	GGGTCCTTTCCAGAATCAGAAACACCGCGGGGCGGCAGACGCGCAAAA<br>ACGCGCTATTTATGACGCTTTCCGGGAAAGGGTACACCACCACCACTT<br>GATTAATATTTAATCGCATTATTAAGGTAACGTTATGAATAATAAAGA<br>TTGTTTTGTTAGTCAGCAGGAAATAGCAGAACATTTCAAGGTCAACAG<br>AACCACTATTCGCGCATGGACCAAACAGGGCATGCCGTATCTTGATGC<br>GGATCGCGGAAAGTCCGGCGGTTATCACATCGGACACACATTATTCTG<br>GTGCATGGGTAAAAGTCATCTTGATGCTATTGAGTACCACG
E. coli HH29S<br>[NZ_VNXH01000006.1]<br>PICI-C	RppC {153} [WP_160513534.1]<br>(*rpp* Range: 193800-195500)<br><br>MSEKEFFLSQQEIADQFGVDRTTVRAWTKRGLPFIEGDK<br>GKPGRYQLGHVLFWVRGQEGLKELGMTGELHPLDCIMHS<br>REIMLSMVGEEEDKQEYEKKFNKGLEIYGYSPDEIAQAR<br>GRAQGIEIGRELTLKRLKKHTNENKKKRKLIRQNDT	GGGTCCTTTCCGGCGATCCGAAAGGTTACGGGGCGGCAGACGCGCAAA<br>AAAGCGCTATTTATGACGCTTTCCGGGAAAGGGTACACCACCACCACT<br>TGATTAATATTCAACCAGTAATTATTGGTAACATTATGAGTGAAAAAG<br>AATTTTTCTTAAGCCAGCAGGAAATAGCAGATCAATTTGGAGTAGACA<br>GGACAACTGTCAGGGCATGGACTAAACGCGGTTTGCCATTCATTGAAG<br>GAGATAAAGGAAAGCCGGGGCGCTACCAGTTAGGACATGTTCTTTTTT<br>GGGTAAGAGGACAGGAAGGACTCAAAGAACTGGGTATGACTG
Pluralibacter gergoviae<br>MGH173<br>[NGRT01000001.1]<br>PICI-G	RppG {139} [OUF48096.1]<br>(*rpp* Range: 2872558..2872977)<br><br>MKGKSFFVSQQDIAEHFGVNRTTIRTWTKQGMPYLEADR<br>GKPGGYHIGHVLWWSMGKRRFEEIEFQGDTSALEKIMFA<br>RLISSELDGPYGEEFERKFDESLLIYGYTLEDVSMARNK<br>MAGFRAGWSHRVAARPNGNSVT	GGGTCCTTTCTGGTGATCCGGCGCGTTACGGGGCGACGACCTCACAGG<br>ATTCCGCTACATATGAAATTTTCTGGGGATACCACACCACCACCACTC<br>CTTGATTAAAAAATAATCAGATGGAAAAGGTAATGTAATGAAGGGTAA<br>GAGTTTTTTTGTAAGTCAGCAGGACATTGCTGAGCATTTCGGCGTAAA<br>CAGAACCACGATCCGCACCTGGACTAAACAGGGCATGCCCTATCTGGA<br>GGCCGATCGTGGGAAGCCCGGTGGTTATCACATTGGCCACGTCTTGTG<br>GTGGAGTATGGGTAAAAGACGCTTTGAAGAAATCGAGTTTCA
Phage λ<br>[NC_001416]	TerS-λ {181} [NP_040580.1]<br>(*Nu1* range: 191-736)<br><br>MEVNKKQLADIFGASIRTIQNWQEQGMPVLRGGGKGNEV<br>LYDSAAVIKWYAERDAEIENEKLRREVEELRQASEADLQ<br>PGTIEYERHRLTRAQADAQELKNARDSAEVVETAFCTFV	GGGTCCTTTCCGGTGATCCGACAGGTTACGGGGCGGCGACCTCGCGGG<br>TTTTCGCTATTTATGAAAATTTTCCGGTTTAAGGCGTTTCCGTTCTTC<br>TTCGTCATAACTTAATGTTTTTATTTAAAATACCCTCTGAAAAGAAAG<br>GAAACGACAGGTGCTGAAAGCGAGGCTTTTTGGCCTCTGTCGTTTCCT<br>TTCTCTGTTTTTGTCCGTGGAATGAACAATGGAAGTCAACAAAAAGCA

	LSRIAGEIASILDGLPLSVQRRFPELENRHVDFLKRDII<br>KAMNKAAALDELIPGLLSEYIEQSG	GCTGGCTGACATTTTCGGTGCGAGTATCCGTACCATTCAGAACTGGCA<br>GGAACAGGGAATGCCCGTTCTGCGAGGCGGTGGCAAGGGTAA
Phage N15<br>[NC_001901.1]	TerS-N15 {168} [1261640]<br>(*1* range = 89-595)<br><br>MAVLLNKSDMASSLGISVQAFDKWGVQPVEKRGREVFFD<br>VRSVVDNRLENQSRKLQPSPGDDDDGVNIDYERWRLTRA<br>NADAAELANEKKRREVVETAFCTFVLSRIAAEISSILDG<br>IPLSMQRRFPELENRHIEFLKGDVIKAMNKAAALDERIP<br>GLLNDYIDQSGS	GGGTCCTTTCCGGCAATCCGCCCTGTTACGGGGCGGCGTCCGCGCAGA<br>TTCTCGCTATATATGAAAAATTTGGACATGAGGTTGTTGTTTAATCAT<br>TGCTGAAAACTGGTGAGGGAATATGGCCGTGTTATTGAATAAATCTGA<br>TATGGCTTCCTCGCTGGGAATATCCGTCCAGGCCTTTGATAAATGGGG<br>TGTCCAGCCTGTAGAGAAGAGGGGTCGTGAAGTCTTCTTTGATGTTCG<br>GTCAGTCGTAGATAACCGGCTCGAAAACCAGAGCAGAAAACTACAACC<br>ATCTCCGGGTGACGACGATGATGGTGTCAATATTGATTATGA
